# Improvement of synaptic plasticity and cognitive function in RASopathies—a monocentre, randomized, double-blind, parallel-group, placebo-controlled, cross-over clinical trial (SynCoRAS)

**DOI:** 10.1186/s13063-023-07392-z

**Published:** 2023-06-06

**Authors:** Nikolai H. Jung, Silvia Egert-Schwender, Beate Schossow, Victoria Kehl, Ute Wahlländer, Louisa Brich, Viktoria Janke, Christiane Blankenstein, Martin Zenker, Volker Mall

**Affiliations:** 1 Social Pediatrics, School of Medicine, Technical University of Munich, Munich, Germany; 2 Münchner Studienzentrum, School of Medicine, Technical University of Munich, Munich, Germany; 3 Institut for General Medicine, Ludwig-Maximilians-University Munich, Munich, Germany; 4 Institute of Human Genetics, University Hospital Magdeburg, Magdeburg, Germany

**Keywords:** RASopathies, Neurofibromatosis type 1, Noonan syndrome, Transcranial magnetic stimulation, Synaptic plasticity, Attention, Lovastatin, Lamotrigine

## Abstract

**Background:**

Cognitive impairment is a common medical issue in rat sarcoma (RAS) pathway disorders, so-called RASopathies, like Neurofibromatosis type 1 (NF1) or Noonan syndrome (NS). It is presumed to be caused by impaired synaptic plasticity. In animal studies, pathway-specific pharmacological interventions with lovastatin (LOV) and lamotrigine (LTG) have been shown to improve synaptic plasticity as well as cognitive function. The aim of this clinical trial is to translate the findings of animal studies to humans and to probe the effect of lovastatin (NS) and lamotrigine (NS and NF1) on synaptic plasticity and cognitive function/alertness in RASopathies.

**Methods:**

Within this phase IIa, monocentre, randomized, double-blind, parallel-group, placebo-controlled, cross-over clinical trial (syn. SynCoRAS), three approaches (approaches I–III) will be carried out. In patients with NS, the effect of LTG (approach I) and of LOV (approach II) is investigated on synaptic plasticity and alertness. LTG is tested in patients with NF1 (approach III). Trial participants receive a single dose of 300 mg LTG or placebo (I and III) and 200 mg LOV or placebo (II) daily for 4 days with a cross-over after at least 7 days. Synaptic plasticity is investigated using a repetitive high-frequency transcranial magnetic stimulation (TMS) protocol called quadri-pulse theta burst stimulation (qTBS). Attention is examined by using the test of attentional performance (TAP). Twenty-eight patients are randomized in groups NS and NF1 with *n* = 24 intended to reach the primary endpoint (change in synaptic plasticity). Secondary endpoints are attention (TAP) and differences in short interval cortical inhibition (SICI) between placebo and trial medication (LTG and LOV).

**Discussion:**

The study is targeting impairments in synaptic plasticity and cognitive impairment, one of the main health problems of patients with RASopathies. Recent first results with LOV in patients with NF1 have shown an improvement in synaptic plasticity and cognition. Within this clinical trial, it is investigated if these findings can be transferred to patients with NS. LTG is most likely a more effective and promising substance improving synaptic plasticity and, consecutively, cognitive function. It is expected that both substances are improving synaptic plasticity as well as alertness. Changes in alertness may be a precondition for improvement of cognition.

**Trial registration:**

The clinical trial is registered in ClinicalTrials.gov (NCT03504501; https://www.clinicaltrials.gov; date of registration: 04/11/2018) and in EudraCT (number 2016–005022-10).

**Supplementary Information:**

The online version contains supplementary material available at 10.1186/s13063-023-07392-z.

## Background

Disorders of the rat sarcoma (RAS) pathway, so-called RASopathies, such as Noonan syndrome (NS), neurofibromatosis type 1 (NF1), Costello syndrome (CS), LEOPARD syndrome and the cardio-facio-cutaneous syndrome (CFC), share common clinical and cognitive deficits that range from poor motor coordination, learning and attention deficits to severe mental retardation [1]. Cognitive impairment is a common medical issue in these patients and variable in its expression. It is presumed to be caused—at least in part—by impaired synaptic plasticity due to dysregulated RAS signalling in neurons [1].

The pathophysiology of cognitive deficits in RASopathies has been studied using a mouse model of NF1. Mice heterozygous for a null mutation of the NF1 gene (Nf1 + / −) have spatial learning problems. It has been shown that hyperactivation of the RAS pathway in these mice leads to an impairment of synaptic plasticity, and pharmacological or genetic approaches that reverse the enhancement in RAS-MAPK (rat sarcoma–mitogen-activated protein kinases) signalling in Nf1 + / − mice also reverse their synaptic plasticity and spatial learning deficits [2, 3]. It is assumed but has not been shown, so far, that the activating mutations in agonists of the RAS-MAPK signalling pathway leading to NS, CFC and CS basically have similar impacts on neuronal function and synaptic plasticity as the loss-of-function in an agonist such as in NF1. In animal studies, pathway-specific pharmacological interventions with lovastatin (LOV) have been shown to improve synaptic plasticity as well as cognitive function [4, 5].

In humans, deficits in synaptic plasticity have been shown in patients with NF1 [6] as well as in patients with NS [7] which were associated with attention deficits [6], reviewed in [8]. In patients with NF1, we have demonstrated in a placebo-controlled, short-term intervention that RAS pathway modulation by LOV reduces gamma-aminobutyric acid (GABA)ergic inhibition and improves synaptic plasticity as well as attention [6, 8]. Hence, we were able to translate the results from animal studies to humans with NF1. This principle of a pathway-specific pharmacological intervention restoring impaired synaptic plasticity and improving cognitive function has been demonstrated in placebo (PLC) controlled intervention with LOV for 4 days [6].

The study of Mainberger et al. [6] also revealed the mechanism of impaired synaptic plasticity in humans with NF1 (*n* = 11) which was associated with increased GABAergic inhibition. The mechanism that GABA is regulating synaptic plasticity has been described previously as “gatekeeping mechanism” [9]. Decreasing intracortical inhibition by LOV was associated with an increase in synaptic plasticity in patients with NF1 [6]. The central role of intracortical inhibition regulating synaptic plasticity in NF1 has recently been certified by animal studies revealing a new option of pharmacological intervention with Lamotrigine (LTG) [5]. The authors have previously demonstrated that LTG is a potent modulator of synaptic plasticity in healthy humans [10].

It is expected and we hypothesize that both substances (LTG and LOV) improve synaptic plasticity as well as alertness in patients with NS and NF1. Changes in alertness may be a precondition for improvement of cognition. The results may contribute to the treatment of patients on an individual level as well as a basis for the initiation of larger multicentre clinical trials.

## Methods

### Aim of the study

The aim of this study is to investigate changes in synaptic plasticity and alertness in patients with RASopathies, namely NS and NF1, after the application of lovastatin (to NS patients) and lamotrigine (to NS and NF1 patients).

### Design

**Fig. 1:**
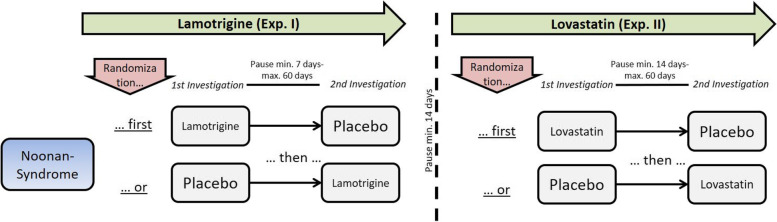
Graphical flowchart of the study design for NS (experiment I and II). In experiment I, patients with Noonan syndrome first receive lamotrigine (LTG) or placebo (PLC) with a minimum pause of 7 days and maximum of 60 days. In experiment II, lovastatin (LOV) or PLC with a minimum pause of 7 days and maximum 60 days. Between experiments, there will be a pause of at least 14 days

**Fig. 2:**
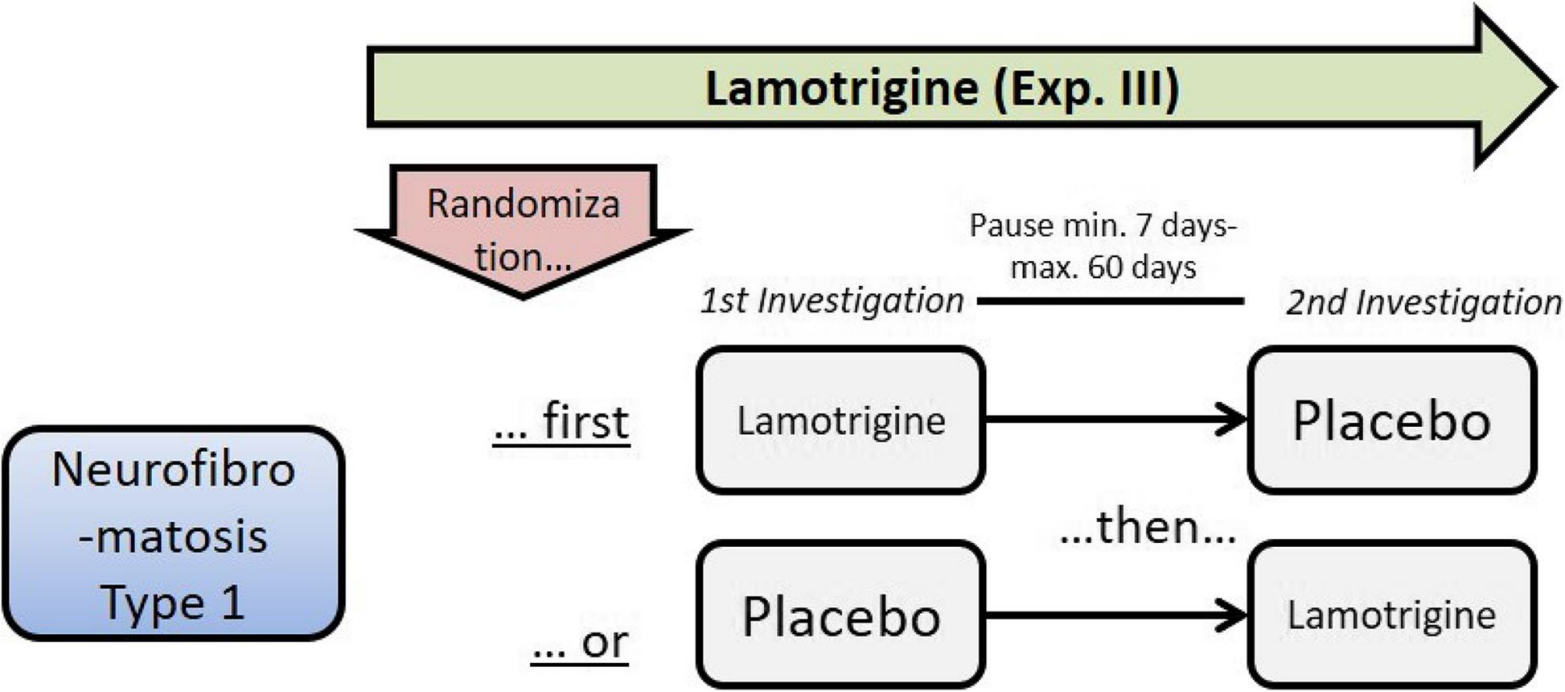
Graphical flow-chart of the study design for NF1 (experiment III). In experiment III, patients with NF1 first receive lamotrigine (LTG) or placebo (PLC) with a minimum pause of 7 days and maximum of 60 days

The SynCoRAS study is a monocentre, randomized, double-blind, parallel-group, placebo-controlled, cross-over clinical trial phase IIa. The trial design is shown in Figs. 1 and 2. The clinical trial has been approved before trial commencement by the local ethics committee (vote 461/17 Af dated 13–02-2018, with the last amendment No. 3 dated 12–11-2020) and by the German medical regulatory authorities (Federal Institute for Drugs and Medical Devices (Bundesinstitut für Arzneimittel und Medizinprodukte (BfArM)). Prior start of recruitment the clinical trial was registered in ClinicalTrials.gov (https://www.clinicaltrials.gov) as well as in the European Union (EU) Clinical Trials Register (EudraCT-Nr.: 2016–005022-10).

### Trial site

Within the monocentric setting, the trial site is located at the Department of Social Pediatrics, School of Medicine, Technical University of Munich. Sponsor-Delegated Person is Prof. Dr. med. Volker Mall.

### Study population

Inclusion criteria are defined as follows: (1) Group 1: NS, Group 2: NF1 (both genetically assured); (2) age ≥ 16 years; (3) signed informed consent of the adolescent (16–17 years of age) who is capable to give his consent and understand the aim and rationale of the study and of the legal guardian; (4) signed informed consent of persons who are ≥ 18 years old and capable to give their consent. In case of doubts, an independent medical practitioner will evaluate the capacity to consent; (5) male and female participants who are not capable of bearing children or who use a method of contraception that is medically approved by the health authority of the respective country.

Exclusion criteria are (1) epilepsy, (2) participants taking medication with known central nervous system (CNS) effects, (3) severe mental retardation, (4) side effects during previous medication with and contraindications for LTG and/or LOV and/or transcranial magnetic stimulation (TMS), (5) psychiatric diseases, (6) previous history of allergic reactions with LTG and LOV medications, (7) potentially unreliable patients, (8) patients who are not suitable for the study in the opinion of the investigator, (9) pregnancy (incl. positive urine pregnancy test) and (10) persons who are incapable of giving consent or do not understand the aim or rationale of the study.

### Interventions

Participants are randomized into either one out of the three approaches (exp. I–III). Three randomizations take place—one for each experiment. Patients are randomly assigned to receive either first verum then placebo, or vice versa. The randomization lists were created using RANCODE professional 2015. Randomization is performed block-wise. The randomization list was provided to the pharmacy for blinding, labelling and distribution of trial medication. For the case of emergency unblinding, a second set of sealed envelopes includes the information on the type of medication for each randomization number and is stored at the trial site. The integrity of the envelopes is monitored until end of study.

#### Experiment I—Lamotrigine (LTG) in patients with NS

Participants receive 300 mg single dose LTG or placebo 2 h before TMS, corresponding to the peak plasma time of LTG [11]. After at least 7 (up to 60) days, a cross-over takes place: participants receive placebo or 300 mg single dose LTG 2 h before TMS (Fig. 1).

#### Experiment II—Lovastatin (LOV) in patients with NS

Participants receive 200 mg LOV or placebo daily for 4 days prior to TMS. On the day of TMS, the medication is given 3 h before starting TMS corresponding to the peak plasma concentration of LOV [12]. After at least 14 (up to 60) days, a cross-over takes place: participants receive placebo or 200 mg LOV daily for 4 days prior to TMS (Table 1, Additional file 1). On the day of TMS, the medication is given 3 h before starting TMS.

**Table 1:** Schedule of procedures and visits patient group 1, NS

*Study overview medication*	**Base-line (or day 1)**	**Visit 1 (day 1)** **D1**	**Visit 2 (min. 7—60 D after V 1)** **D8–61**	**Visit 3 (min. 14–60 D after V2)** **D22–121**	**Visit 4 (3 D after V3)** **D25–124**	**Visit 5 (min. 14–60 D after V4)** **D39–184**	**Visit 6 (3 D after V5)** **D42–187**
		**Exp. I: Lamotrigine (LTG) vs. placebo, single dose (with cross-over)**		**Exp II: Lovastatin (LOV) vs. placebo 4 days (with cross-over)**			
Informed consent	**X**						
Eligibility criteria	**X**						
Demographics	**X**						
Medical/surgical history	**X**						
Vital signs	**X**	**X**	**X**	**X**		**X**	
Pregnancy test^a^	**X**			**X**			
Randomization		**X**		**X**			
Exp. I: LOV or PLC) Four days				**X**	**X**	**X** **(cross-over)**	**X**
Exp. II: LTG or PLC) Single dose		**X**	**X** **(cross-over)**				
TMS (electromyography (EMG) integrated)^b^		**X**	**X**		**X**		**X**
TAP		**X**	**X**		**X**		**X**
Concomitant medication	**X**	**X**	**X**	**X**	**X**	**X**	**X**
AE/SAE		**X**	**X**	**X**	**X**	**X**	**X**

^a^To be performed after having obtained informed consent; if baseline is less than 5 days prior to V1, a second pregnancy test is not necessary

^b^TMS to be performed 3 h after intake of LOV and 2 h after intake of LTG

#### Experiment III—Lamotrigine (LTG) in patients with NF1

Participants receive 300 mg single dose LTG or placebo 2 h before TMS [11]. After at least 7 (up to 60) days, a cross-over takes place: participants receive placebo or 300 mg single dose LTG 2 h before TMS (Table 2, Additional file 1).

**Table 2:** Schedule of procedures and visits patient group 2, NF1

*Study overview medication*	**Baseline visit (or day 1)**	**Visit 1 (day1)** **D1**	**Visit 2 (7–60 days after V 1)** **D8–61**
		Exp. III: Lamotrigin (LTG) or Placebo	
Informed consent	**X**		
Eligibility criteria	**X**		
Demographics	**X**		
Medical/surgical history	**X**		
Vital signs	**X**	**X**	
Pregnancy test^a^	**X**	**X**	
Randomization		**X**	
Exp. III: LTG or PLC) Single dose		**X**	**X** **(cross-over)**
TMS (EMG integrated)^a^		**X**	**X**
TAP		**X**	**X**
Concomitant medication	**X**	**X**	**X**
AE/SAE		**X**	**X**

^a^To be performed after having obtained informed consent; if baseline is less than 5 days prior to V1, a second pregnancy test is not necessary

^b^TMS to be performed 2 h after intake of LTG

Experiments I and II take place with a sufficient time gap on the same set of patients (Fig. 1). It is not possible to conceal treatment in a triple cross-over design in group 1, which is why two separate tests have been planned on group 1.

### Transcranial magnetic stimulation (TMS)

Transcranial magnetic stimulation (TMS) is a standard method used in medical diagnostics and research in humans [13, 14]. The planned investigations promise progressing basic findings regarding the neurophysiological mechanisms of the TMS and of neuronal plasticity in patients with NF1 and NS. Planned neurophysiological investigations with TMS delivered by the PowerMAG quadri-pulse stimulation (QPS) device (Mag & More GmbH, Munich) bear only very little risks and have been used by the investigators before [15]. An increased susceptibility to seizures, which can be observed with high-frequency, above-threshold TMS, was not seen so far using the planned stimulation protocols [15]. The clicking noise, which appears using transcranial magnetic stimulation, does not cause any adverse effect. The examination is not painful. Some participants are not used to the involuntary muscle contraction, which appears under magnetic stimulation, and sometimes this is felt as an awkward sensation [14]. However, experience has shown a fast adaption to these contractions. Participants are informed about this beforehand.

**Fig. 3:**
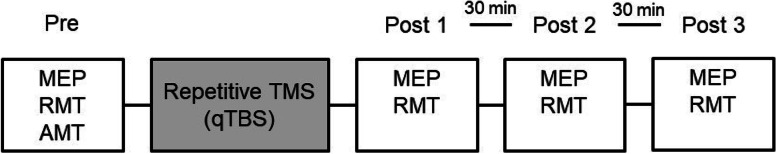
Timeline of TMS measurement. After pre-measurements, patients (NS and NF1) will receive a quadri-pulse theta burst stimulation (qTBS) to evaluate changes in cortico-spinal excitability. Motor evoked potentials (MEP) and resting motor threshold will be monitored 2–5 min, 30 min and 60 min after qTBS. MEP, motor evoked potential; RMT, resting motor threshold; AMT, active motor threshold; qTBS, quadri-pulse theta burst stimulation

No other undesirable side effects are expected during stimulation. The investigators used TMS in patients with NF1 [6] as well as with NS [7] reviewed in [8] to demonstrate deficits in synaptic plasticity which were associated with attention deficits (NF1: [6]). The investigators have a long time experience with TMS and published several manuscripts in international journals [10, 15–17]. The study procedure contains pre-measurements before interventional, repetitive TMS using the quadri-pulse theta burst stimulation protocol (qTBS) [15] and three (Post 1–3) follow-up measurements (Fig. 3). Changes in motor-evoked potentials (MEP), referring to the model of long-term potentiation (LTP)-like plasticity, as primary outcome measure, are monitored until 1 h after interventional TMS, conducted by the qTBS protocol. Changes in local excitability are investigated by the resting motor threshold.

### Test for Attentional Performance (TAP)

The computerized Test for Attentional Performance (TAP) battery is a widely used tool in the systematic assessment of attention [6]. Attention skills of the participants are evaluated by conducting the Alertness, Visual Scanning, GoNogo and Incompatibility task. These four subtests of the TAP were selected because of a strong analogy to attention deficit responsive testing in patients with NF1. The Alertness test is a visual reaction time task to a presented visual target stimulus (cross) with or without previous acoustic warning. The Visual Scanning test requires accuracy and performing speed while detecting the target in a field of similar figures. The GoNogo test assesses the ability to discriminate between a target and a similar but irrelevant non-target, where participants have to respond to the target and inhibit their response to the non-target. In the Incompatibility test, participants have to detect the direction of a simple arrow (right/left) by pressing a button on the left or right side regardless on which side of the screen (right/left) the arrow appears.

### Endpoints and endpoint rationale

The *primary endpoint* for each experiment is the difference between the amplitude of the motor evoked potential (MEP) elicited with transcranial magnetic stimulation (TMS, measured at three time points after interventional TMS for each investigation) after placebo and after medication (LTG and LOV).

Changes in MEP are a well-established and safe method to probe changes in cortico-spinal excitability in humans [18]. MEPs are elicited by TMS using a figure-of-eight coil with an outer diameter of 100 mm centred tangentially on the scalp over the primary motor cortex (M1) of the nondominant hand with its handle pointing in a posterior direction and laterally at an angle of approximately 45° away from the midline. MEPs are recorded from the abductor pollicis brevis (APB) muscle at rest by surface electromyography (EMG) using silver/silver chloride electrodes with a surface area of 263 mm^2^ (AMBU, Ballerup, Denmark) mounted in belly-tendon recording technique. Data are band-pass filtered (20–2000 Hz) and amplified using an Ekida DC universal amplifier (EKIDA GmbH, Helmstadt, Germany), digitized at 5 kHz sampling rate using a MICRO1401*mkII* data acquisition unit (Cambridge Electronic Design Ltd, Cambridge, UK) and stored on a standard personal computer for online visual display and later offline analysis using Signal Software version 5 (CED Ltd, UK). MEP size is determined by measuring the two highest peaks of opposite polarity. Twenty trials will be recorded and then averaged for each point of investigation as described elsewhere [15, 16]. Resting motor threshold is recorded to probe changes in local cortical excitability [19].

The *secondary endpoints* for each experiment include the difference between the neuropsychological testing of attention by the TAP and differences in short interval cortical inhibition (SICI) after placebo and after medication (LTG and LOV) [6, 20]. Another endpoint is the comparison of LTG and LOV effects on synaptic plasticity and attentional performance in the NS group. The TAP is a well-established tool and used to measure the influence of LTG and LOV on attention [7, 20]. We previously demonstrated that LOV leads to an amelioration of attentional performance in humans with NF1 [6]. Consequently, we hypothesized that this would also lead to better attention in patients with NS and that LTG ameliorates attention in NF1 and NS.

For SICI, a subthreshold conditioning stimulation is delivered 2, 3 and 5 ms before a test stimulus [6]. It has been demonstrated before that an increased inhibition is the most prominent factor in mice with NF1 to prevent synaptic plasticity [5]. The authors have previously demonstrated that LTG is a potent modulator of synaptic plasticity in healthy humans [10]. Decreasing intracortical inhibition by LOV was associated with an increase in synaptic plasticity in patients with NF1 [6]. The central role of intracortical inhibition regulating synaptic plasticity in NF1 has recently been certified by animal studies revealing a new option of pharmacological intervention with LTG [5]. We have previously shown that LOV leads to a disinhibition in patients with NF1 and, therefore, hypothesized that this would be a key factor to normalize cortico-spinal excitability in patients with NF1 and NS.

*Safety measures* include documentation of blood pressure and heart frequency prior to the intervention as well as monitoring of EMG activity during TMS intervention and documentation of adverse events (AE), severe adverse events (SAE) and suspected unexpected serious adverse reactions (SUSAR) following established definitions and legal requirements. The intensity of AEs is defined according to the common terminology criteria for adverse events (CTCAE Version 4.0, https://www.eortc.be/services/doc/ctc/CTCAE_4.03_2010-06-14_QuickReference_5x7.pdf).

### Analyses and statistics

This is a series of three experiments in a double-blind, placebo-controlled, randomized, cross-over design. Exp. I: LTG vs. placebo on group 1, Exp. II: LOV vs. placebo on group 1, Exp. III: LTG vs. placebo on group 2.

Sample size: Sample size calculation was done using the primary endpoints, nQuery Advisor, and expected means of MEP amplitudes and standard deviation (SD) for the different measurements from Mainberger et al. [7].

**Table:** 

	Timepoint 1 (T1)	Timepoint 2 (T2)	Timepoint 3 (T3)
**Lovastatin LTP (increase in mean MEP) data (Mainberger et al. **[7]**) with common SD of 0.5**			
Healthy controls	1.440	1.600	1.710
NF1	0.920	0.970	0.980
Difference	0.520	0.630	0.730
**Experiments I and II**			
Local α	0.025	0.013	0.008
Power with 14 patients	89%	95%	97%
Power with 12 patients	82%	89%	94%
**Experiment III**			
Local α	0.050	0.025	0.017
Power with 14 patients	94%	97%	99%
Power with 12 patients	90%	94%	97%

An increase in MEP amplitudes over time is considered to refer to the concept of long-term potentiation (LTP)-like plasticity as it has been demonstrated in Mainberger et al. [7] as well. Twelve patients per disease entity will be enough to provide appropriate power to all planned primary endpoint tests (two-sided paired samples *t*-tests) with a global significance level of 5% and adjusted local significance levels using the Bonferroni-Holm procedure. In order to account for some unobtainable data and drop outs, the sample size will be increased to 14 patients per disease (28 trial participants in total).

Analysis sets: For each experiment, there will be a separate analysis set defined, as it is possible that a patient is suitable for analysis in only one of the first two experiments. All analyses will be performed on the full analysis set (FAS-I, FAS-II, FAS-III), consisting of all patients who delivered a full set of MEP measurements within the corresponding experiment. Since all measurements will take place within a very short time per patient, it is expected that the data will be either complete or entirely missing.

Primary endpoint: The primary endpoint analyses will be performed in three separate testing procedures. The global significance level will be 5%. Since experiments I and II are done on the same set of patients, the significance level for those two experiments will each be 2.5%. The significance level of experiment III will be 5%. The primary endpoint analysis will consist of three series of three paired samples two-sided* t*-tests, comparing MEP under verum vs. placebo at the three measurement time points. The local significance level will be adjusted using the Bonferroni-Holm procedure and will be as follows for the ordered by *p*-value tests:

**Table:** 

Experiment	Test number	Local significance level
I and II	1	0.025
I and II	2	0.013
I and II	3	0.008
III	1	0.050
III	2	0.025
III	3	0.017

Secondary endpoints and safety: Analyses of baseline and safety data and secondary endpoints will be done using appropriate descriptive statistics and paired sample tests for difference between the two study groups. All tests will be two-sided with an exploratory significance level of 5%. No adjustment for multiple comparisons will be done.

### Organizational framework

Organizational/regulatory project management, safety management, monitoring and data management are performed by the Münchner Studienzentrum (MSZ), Technical University of Munich, School of Medicine. An independent safety monitoring board (SMB) is established. The underlying principles for the SMB are ethical and safety aspects for the patients. The SMB examines whether the conducting of the study is still ethically justifiable, whether safety of the patients is ensured and whether the process of the study is acceptable. For this, the SMB is informed regularly about patient recruitment and observed safety advents. Serious adverse events (SAEs) are recorded in a study-specific safety form and transferred into a safety database by the safety management, which processes further SAE documentation in written form to the SMB, the Investigator, the ethics committee and regulatory authority (BfArM) according to applicable law.

All study procedures agree with the guidelines of Good Clinical Practice (GCP) of the International Council on Harmonization of Technical Requirements for Pharmaceuticals for Human Use (ICH) and the principles of the Declaration of Helsinki. All participating investigators agreed to adhere to the instructions and procedures described in the study protocol and thereby to adhere to the principles of ICH-GCP. All members of the safety board are independent from the sponsor. The current approved protocol version is 5.0 (amendment 3, 21.09.2020). All protocol versions are available in German language at nikolai.jung@tum.de.

## Discussion

The SynCoRAS study is targeting impairments in synaptic plasticity and cognitive impairment, one of the main health problems of patients with RASopathies. This has been done by the authors successfully for Lovastatin in NF1 before [6]. The obtained results will be transferred to patients with Noonan syndrome which demonstrated a reduced plasticity before as well [7]. Lamotrigine is most likely a more effective and promising substance improving synaptic plasticity and, consecutively, cognitive function [5, 10]. It is expected that both substances are improving synaptic plasticity as well as alertness. Changes in alertness may be a precondition for improvement of cognition. The strength of this project may be the short-term translation of results of basic science to human application and the provision of insights in pathophysiological mechanism. Results will be brought to practice through the tight connection to the large clinical centre for patients with cognitive impairment. Results might be used for the treatment of patients on an individual level as well as for the initiation of large clinical trials.

### Risk/benefit analysis

There are known side effects of lovastatin (LOV) and lamotrigine (LTG). LOV and LTG are used in an off-label context and in a higher dosage as recommended in the Summary of Product Characteristics (SMPC) of the medicinal products. However, all side effects of the medication (LOV and LTG) have been described after longer application whereas in the present study patients will receive LOV only four times and LTG only one time. In one patient receiving LTG, a mild rash following the administration reported has been reported [21]. In an own placebo-controlled, randomized, double-blind study using the same single dose of LTG (300 mg), no side effects, apart from mild sedation/dizziness and lacrimation have been reported in 26 young healthy adults [10]. LTG has been well tolerated in another TMS study using dosages up to 325 mg [11]. Severe skin reactions such as Stevens-Johnson syndrome, toxic epidermal necrolysis, drug rash with Eosinophilia and systemic symptoms (DRESS) syndrome or aseptic meningitis occurring 8 weeks after long-term application of LTG have never been mentioned after single-dose application. These adverse events can be excluded to the actual state of knowledge.

A 4-day course of LOV has been used in an own study in *n* = 11 patients with NF1 and is not expected to provoke any side effects [6]. It has been shown that doses of 200 mg of LOV were proven to be save in adult human volunteers [22]. Moreover, accidental overdoses up to 6 g have been tolerated with no specific symptoms and recovery without sequelae. Adverse events of LOV occur after longer application (> 1–2 weeks). Overall, the treatment with LTG and LOV in the context of the present study is not expected to provoke any side effects. A randomized, placebo-controlled long-term study investigated the effect of simvastatin, another 3-hydroxy-3-methyl-glutaryl-coenzyme A (HMG-CoA) reductase inhibitor, on cognition in children with NF1 [23]. This study did not find effects of simvastatin on cognition in children with NF1 [23]. In the present study, LOV will be used to investigate its effects on synaptic plasticity and attentional performance in patients with NS. We previously demonstrated in a randomized, placebo-controlled trial effects of LOV on synaptic plasticity and attentional performance in patients with NF1 in short-term use [6]. Moreover, another study demonstrated effects of lovastatin in children with NF1 undergoing a dose escalation protocol for 3 months on verbal and nonverbal memory [24]. This may highlight potential pharmacological differences of LOV and simvastatin.

The results of the present study may help to design long-term studies focusing on the therapeutic effects of LTG on synaptic plasticity and cognition in patients with NF1. It is intended to publish the results in an international scientific journal. The inclusion of minors from the age of 16 years is not considered to increase the risk of potential side effects since persons are considered to be grown-up at this age from a medical point of view. We do not expect significant differences in body weight or in the metabolism of both medications (LTG and LOV) in adolescents aged 16 years why an adaption of the dosage is not necessary. LTG is a safe and commonly used medication in the treatment of seizures/epilepsy in children and adolescents. For medications (LTG and LOV), the risk of side effects after a single dosage (LTG) and a 4-day course of LOV is not considered to be increased in adolescents. Moreover, we estimate the benefit of an early treatment of attention deficits with the medication to be higher than the risk of the treatment in this study. Side effects associated with LTG or LOV are closely monitored and handled as described above. In case of acute threatening of the patients, treatment will be handled as described in the emergency plan of the hospital.

### Trial status

The current protocol version number is “Final 5.0 AM 3.0”, date 9/21/2020. Recruitment finished on 3/30/2023 prematurely due to unforeseen difficulties in recruitment of patients. Recruitment was still open on initial submission of the manuscript. Recruitment began in March 2018, and an actual number of 16 participants have been enrolled. A large panel including the sponsor-delegated person, the head of German Network of RASopathy Research (GeNeRARe) and the Sponsors representative for risk-based quality management decided to prematurely close the recruiting and to terminate the study in retrospect on 2/20/2023 due to the last patient last visit (LPLV) taking place on 2/9/2023.

## Conclusion

In summary, we aim to investigate the effects of LTG and LOV on synaptic plasticity and cognitive function in patients with NS and LTG in patients with NF1. The results may provide significant information about the effectiveness and the mode of action of these interventions in humans and may help to design larger multicentre clinical trials. The strength of the project may be the short-term translation of results of basic science in the setting of a large hospital with a high reputation in medical care of patients with cognitive disorders.

## Supplementary Information

**Additional file 1.** SPIRIT 2013 Checklist.

## Data Availability

Anonymized datasets that will be analysed during the current study will be available from the corresponding author on reasonable request.

## Abbreviations

AE: Adverse event
APB: Abductor pollicis brevis
BfArM: Bundesinstitut für Arzneimittel und Medizinprodukte
BW: Body weight
CFC: Cardio-facio-cutaneous syndrome
CNS: Central Nervous System
CRF: Case Report Form
CS: Costello syndrome
CTCAE: Common terminology criteria for adverse events
DRESS: Drug rash with Eosinophilia and systemic symptoms
GCP: Good Clinical Practice
eCRF: Electronic Case Report Form
EMG: Electromyography
ER: Emergency Room
EU: European Union
FAS: Full analysis set
FPFV: First Patient First Visit
GABA: Gamma-aminobutyric acid
GCP: Good Clinical Practice
HMG-CoA: 3-Hydroxy-3-methyl-glutaryl-coenzyme A
ICH: International Council on Harmonization of Technical Requirements for Pharmaceuticals for Human Use
ITT: Intention to treat
LTG: Lamotrigine
LTP: Long-term potentiation
LPFV: Last Patient First Visit
LPLV: Last Patient Last Visit
LOV: Lovastatin
M1: Primary motor cortex
MAPK: Mitogen-activated protein kinases
MEP: Motor Evoked Potential
MSZ: Münchner Studienzentrum
NS: Noonan Syndrome
NF1: Neurofibromatosis type 1
PP: Per protocol
PLC: Placebo
RAS: Rat sarcoma
SAE: Serious adverse event
SD: Standard deviation
SICI: Short interval cortical inhibition
SMB: Safety monitoring board
SMPC: Summary of Product Characteristics
SUSAR: Suspected unexpected serious adverse reactions
TAP: Test of Attentional Performance
T1-3: Timepoint 1–3
TMS: Transcranial Magnetic Stimulation
QPS: Quadri-pulse stimulation
qTBS: Quadri-pulse theta burst stimulation
